# Frictional Heating During Sliding of Two Layers Made of Different Materials

**DOI:** 10.3390/ma18225088

**Published:** 2025-11-09

**Authors:** Katarzyna Topczewska, Aleksander Yevtushenko, Przemysław Zamojski

**Affiliations:** Faculty of Mechanical Engineering, Bialystok University of Technology, 45C Wiejska Street, 15-351 Bialystok, Poland; a.yevtushenko@pb.edu.pl (A.Y.); p.zamojski@pb.edu.pl (P.Z.)

**Keywords:** friction, heat generation, convective cooling, imperfect thermal contact, temperature

## Abstract

The non-stationary heat problem of friction for two homogeneous layers with imperfect thermal contact and convective heat exchange on the free surfaces is considered. Assuming a constant specific power of friction, an exact solution of the formulated problem is obtained using the Laplace integral transform. The solution is verified by checking the fulfillment of the boundary and initial conditions both in the transform space as well as in the space of the original. Particular solutions are also derived for some specific cases, namely, the perfect thermal contact of friction at large values of the contact heat transfer coefficient and the asymptotic solution at the initial time moments of the heating process. On the basis of developed solutions, numerical analysis was performed in dimensionless form. The influence of the thermal contact conductance, the convective cooling intensity, and the relative layer thickness on the temperature field is investigated. It was established that for Biot number Bi≥50 yields nearly equal surface temperatures.

## 1. Introduction

The need to determine the effective thickness of the thermal layer in various types of structural elements of machines, mechanisms, and devices operating under conditions of short-term thermal interactions is an important aspect of many engineering problems. These include, for example, evaluating the temperature regimes of braking systems [1,2,3], clutches [4,5], and heavily loaded gears [6], as well as investigating surface melting phenomena in friction contacts [7], among others [8]. The effective thickness of the elements of friction, paired based on the concept of effective heat penetration depth, was determined in the article [9]. This concept defines the distance measured along the normal from the surface of friction at which the temperature rise becomes negligible compared to the surface temperature increase. In disc braking systems, the effective thickness of the thermal layer in both the pad (i=1) and the disc (i=2) is determined using the formula $d_{eff,i}=\lambda \sqrt{k_{i}t_{s}}$, where $k_{i}$ is the thermal diffusivity of the i-th material, $t_{s}$ is the time of braking, and λ is the numerical coefficient whose value depends on the physical reasoning underlying the definition of effective thermal layer thickness. For instance, in the case of short-term braking, the coefficient value λ=1.73 was adopted [10].

It has been established that when the effective heat penetration depth $d_{eff,i}$ exceeds the actual thickness $d_{i}$, i=1, 2 of an element, the latter $d_{i}$ is taken as the geometric reference parameter for determining the temperature field. In the opposite case, the effective heat penetration depth $d_{eff,i}$ is used in the calculations. Depending on the relationship between $d_{eff,i}$ and $d_{i}$, the appropriate one-dimensional heat problems of friction are formulated using the schemes of semi-bounded $\left(d_{eff,i}<d_{i}\right)$ or bounded bodies $\left(d_{eff,i}\geq d_{i}\right)$ [11,12,13]. In the latter case, a system of two sliding layers is typically adopted. The pioneering formulation of the heat problem of friction for such a scheme was presented in [14]. It takes into account the conditions of perfect thermal contact on the surface of friction and thermal insulation of the free surfaces of the layers. However, the solution to the problem was obtained only in the space of the Laplace integral transform. Another approach to solving this problem was demonstrated in [15], in which the condition of maintaining the initial temperature on the surfaces of the free layers was assumed. The criteria for thermal stability for the system of two sliding layers were established [16] as well.

Classical models of frictional heating in sliding systems often assume perfect thermal contact [10]. However, this assumption only represents the macroscopic geometry of ideally smooth surfaces and neglects tribological phenomena at the interface. In fact, the contact between sliding solids is imperfect: surface roughness reduces the real contact area relative to the nominal one, leading to temperature discontinuities across the interface. Accurate prediction of temperature fields in a friction zone, therefore, requires consideration of imperfect thermal contact [17,18,19,20]. The formulation of the thermal friction problem for a system of two sliding layers with conditions of imperfect thermal contact of friction (at the constant value of the thermal contact conductance) on the sliding surface and convective heat exchange with the surrounding environment was proposed in [21]. The solution of the appropriate boundary-value heat conductivity problem, with consideration of the heat generation due to friction, was sought in the form of a sum of solutions of the stationary heat conductivity problem with the above-mentioned boundary conditions and the appropriate non-stationary problem with zero boundary conditions and non-homogeneous initial condition. The solution of the latter problem was obtained by the method of separation of variables and was written in such a complicated form that the authors managed to carry out the numerical analysis only for the specific case of perfect thermal contact of friction. In [22], the case of the thermal contact conductance changing over time was considered.

The aim of this study is to obtain an exact solution of the thermal problem of friction for two layers in the formulation proposed in [21], but using the mathematical apparatus of the Laplace integral transform. The solution is obtained in a form that allows for carrying out a comprehensive numerical analysis at any physically justified values of the thermal contact conductance. It is also possible to verify the obtained solution both in the space of transforms and originals. For the first time, an appropriate asymptotic solution is presented at the initial moments of the friction heating process (at small values of the Fourier number).

## 2. Statement of the Problem

**Figure 1:**
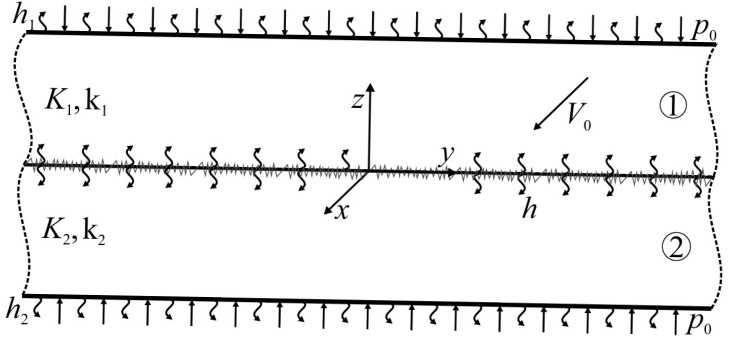
Scheme of friction heating and convective cooling of a two-layer system.

The system of two different homogeneous layers, related to the Cartesian coordinate system Oxyz (Figure 1), is under consideration. The layers are compressed by uniform normal pressure $p_{0}$ on the surfaces $z=d_{1}$ and $z=-d_{2}$. At the initial moment of time t=0 they start to slide with relative constant speed $V_{0}$ in the positive direction of the axis Ox. As a result of friction on the contact surface z=0, heat is generated, and the layers heat up. The following can be assumed:

- The thermal conductivity $K_{l}$ and thermal diffusivity $k_{l}$, l=1, 2 of the layer materials and the coefficient of friction f do not change under the influence of temperature.
- The thermal contact of friction is imperfect, with the thermal contact conductance h>0. This parameter is related to the roughness of contacting surfaces, wherein a decrease leads to a higher thermal contact conductance [23,24].
- The free surfaces $z=d_{1}$ and $z=-d_{2}$ are cooled by convection with the heat transfer coefficients $h_{1}$ and $h_{2}$, respectively.
- The heat losses due to wear are negligible.

Based on the above assumptions, the following thermal problem of friction to find the temperature field T(z,t) in the layers was formulated:

(1) $$\frac{\partial^{2}T(z,t)}{\partial z^{2}}-\frac{1}{k_{1}}\frac{\partial T(z,t)}{\partial t}=0,\;0<z<d_{1},\;t>0,$$

(2) $$\frac{\partial^{2}T(z,t)}{\partial z^{2}}-\frac{1}{k_{2}}\frac{\partial T(z,t)}{\partial t}=0,-d_{2}<z<0,\;t>0,$$

(3) $${\left.{\left.K_{2}\frac{\partial T(z,t)}{\partial z}\right|}_{z=0^{-}}-K_{1}\frac{\partial T(z,t)}{\partial z}\right|}_{z=0^{+}}=q_{0},\;t>0,$$

(4) $${\left.{\left.K_{2}\frac{\partial T(z,t)}{\partial z}\right|}_{z=0^{-}}+K_{1}\frac{\partial T(z,t)}{\partial z}\right|}_{z=0^{+}}-h[T(0^{+},t)-T(0^{-},t)]=0,\;t>0,$$

(5) $${\left.K_{1}\frac{\partial T(z,t)}{\partial z}\right|}_{z=d_{1}}-h_{1}[T_{0}-T(d_{1},t)]=0,\;t>0,$$

(6) $${\left.K_{2}\frac{\partial T(z,t)}{\partial z}\right|}_{z=-d_{2}}-h_{2}[T(-d_{2},t)-T_{0}]=0,\;t>0,$$

(7) $$T(z,0)=T_{0},-d_{2}\leq z\leq d_{1},$$

where $q_{0}=fp_{0}V_{0}$ is the specific power of friction and $T_{0}$ is the initial temperature of the system. Here and further, all values and parameters referring to the upper $0\leq z\leq d_{1}$ and lower $-d_{2}\leq z\leq 0$ layers have lower indices l=1 and l=2, respectively. The symbols $0^{\pm }$ denote the limiting values of the spatial variable z at approaching zero from the positive or negative direction of the Oz axis, respectively.

The following dimensionless variables and parameters were introduced:

(8) $$\zeta =\frac{z}{d_{1}},\;\tau =\frac{k_{1}t}{d_{1}^{2}},\;d^{*}=\frac{d_{2}}{d_{1}},\;K^{*}=\frac{K_{2}}{K_{1}},\;k^{*}=\frac{k_{2}}{k_{1}},\;Bi=\frac{hd_{1}}{K_{1}},\;Bi_{1}=\frac{h_{1}d_{1}}{K_{1}},\;Bi_{2}=\frac{h_{2}d_{2}}{K_{2}},\Theta^{*}=\frac{\Theta }{\Theta_{0}},$$

where $\Theta =T-T_{0}$, $\Theta_{0}=q_{0}d_{1}K_{1}^{-1}$. Taking into account the notations (8), the problem (1)–(7) was written in the form:

(9) $$\frac{\partial^{2}\Theta^{*}(\zeta ,\tau )}{\partial \zeta^{2}}-\frac{\partial \Theta^{*}(\zeta ,\tau )}{\partial \tau }=0,\;0<\zeta <1,\;\;\;\tau >0,$$

(10) $$\frac{\partial^{2}\Theta^{*}(\zeta ,\tau )}{\partial \zeta^{2}}-\frac{1}{k^{*}}\frac{\partial \Theta^{*}(\zeta ,\tau )}{\partial \tau }=0,-d^{*}<\zeta <0,\;\;\;\tau >0,$$

(11) $$K^{*}{\left.\frac{\partial \Theta^{*}(\zeta ,\tau )}{\partial \zeta }\right|}_{\zeta =0^{-}}-{\left.\frac{\partial \Theta^{*}(\zeta ,\tau )}{\partial \zeta }\right|}_{\zeta =0^{+}}=1,\;\;\;\tau >0,$$

(12) $$K^{*}{\left.\frac{\partial \Theta^{*}(\zeta ,\tau )}{\partial \zeta }\right|}_{\zeta =0^{-}}+{\left.\frac{\partial \Theta^{*}(\zeta ,\tau )}{\partial \zeta }\right|}_{\zeta =0^{+}}-Bi\;[\Theta^{*}(0^{+},\tau )-\Theta^{*}(0^{-},\tau )]=0,\;\;\;\tau >0,$$

(13) $${\left.\frac{\partial \Theta^{*}(\zeta ,\tau )}{\partial \zeta }\right|}_{\zeta =1}+Bi_{1}\;\Theta^{*}(1,\tau )=0,\;\;\;\tau >0,$$

(14) $$d^{*}{\left.\frac{\partial \Theta^{*}(\zeta ,\tau )}{\partial \zeta }\right|}_{\zeta =-d^{*}}-Bi_{2}\;\Theta^{*}(-d^{*},\tau )=0,\;\;\;\tau >0,$$

(15) $$\Theta^{*}(\zeta ,0)=0,-d^{*}\leq \zeta \leq 1.$$

The solution of the boundary-value problem of heat conductivity (9)–(15) will be found by using the technique of the Laplace integral transform.

## 3. Solution of the Problem in the Laplace Transforms Space

Using the Laplace integral transform [25]:

(16) $${\bar{\Theta }}^{*}(\zeta ,p)\equiv L[\Theta^{*}(\zeta ,\tau );p]=\int_{0}^{\infty }\Theta^{*}(\zeta ,\tau )\exp(-p\tau )d\tau ,$$

the boundary-value problem (9)–(15) was reduced to the following boundary problem for two ordinary differential equations (ODEs):

(17) $$\frac{d^{2}{\bar{\Theta }}^{*}(\zeta ,p)}{d\zeta^{2}}-p{\bar{\Theta }}^{*}(\zeta ,p)=0,\;0<\zeta <1,$$

(18) $$\frac{d^{2}{\bar{\Theta }}^{*}(\zeta ,p)}{d\zeta^{2}}-\frac{p}{k^{*}}{\bar{\Theta }}^{*}(\zeta ,p)=0,-d^{*}<\zeta <0,$$

(19) $${\left.K^{*}\frac{d{\bar{\Theta }}^{*}(\zeta ,p)}{d\zeta }\right|}_{\zeta =0^{-}}-\;{\left.\frac{d{\bar{\Theta }}^{*}(\zeta ,p)}{d\zeta }\right|}_{\zeta =0^{+}}=\frac{1}{p},$$

(20) $${\left.K^{*}\frac{d{\bar{\Theta }}^{*}(\zeta ,p)}{d\zeta }\right|}_{\zeta =0^{-}}+{\left.\frac{d{\bar{\Theta }}^{*}(\zeta ,p)}{d\zeta }\right|}_{\zeta =0^{+}}-Bi\;[{\bar{\Theta }}^{*}(0^{+},\tau )-{\bar{\Theta }}^{*}(0^{-},\tau )]=0,$$

(21) $${\left.\frac{d{\bar{\Theta }}^{*}(\zeta ,p)}{d\zeta }\right|}_{\zeta =1}+Bi_{1}\;{\bar{\Theta }}^{*}(1,p)=0,$$

(22) $${\left.d^{*}\frac{d{\bar{\Theta }}^{*}(\zeta ,p)}{d\zeta }\right|}_{\zeta =-d^{*}}-Bi_{2}\;{\bar{\Theta }}^{*}(-d^{*},p)=0.$$

The general solution of ODE’s (17) and (18) has the form:

(23) $$\bar{\Theta }(\zeta ,p)=A_{1}(p)\mathrm{sh}(\zeta \sqrt{p})+B_{1}(p)\mathrm{ch}(\zeta \sqrt{p}),\;0\leq \zeta \leq 1,$$

(24) $$\bar{\Theta }(\zeta ,p)=A_{2}(p)\mathrm{sh}\left(-\zeta \sqrt{\frac{p}{k^{*}}}\right)+B_{2}(p)\mathrm{ch}\left(-\zeta \sqrt{\frac{p}{k^{*}}}\right),-d^{*}\leq \zeta \leq 0,$$

where $A_{l}(p)$, $B_{l}(p)$, l=1,2 are the unknown function of the Laplace integral transform parameter p (16). Taking into account the derivatives [26]:

(25) $${\mathrm{sh}}^{\prime }(x)=\mathrm{ch}(x),\;{\mathrm{ch}}^{\prime }(x)=\mathrm{sh}(x),$$

from solutions (24) and (25), it was found that:

(26) $$\frac{d{\bar{\Theta }}^{*}(\zeta ,p)}{d\zeta }=\sqrt{p}[A_{1}(p)\mathrm{ch}(\zeta \sqrt{p})+B_{1}(p)\mathrm{sh}(\zeta \sqrt{p})],\;0\leq \zeta \leq 1,$$

(27) $$K^{*}\frac{d{\bar{\Theta }}^{*}(\zeta ,p)}{d\zeta }=-\varepsilon \sqrt{p}\left[A_{2}(p)\mathrm{ch}\left(-\frac{\delta }{d^{*}}\zeta \sqrt{p}\right)+B_{2}(p)\mathrm{sh}\left(-\frac{\delta }{d^{*}}\zeta \sqrt{p}\right)\right],-d^{*}\leq \zeta \leq 0,$$

where

(28) $$\delta =\frac{d^{*}}{\sqrt{k^{*}}},\;\varepsilon =\frac{K^{*}}{\sqrt{k^{*}}},$$

ε is the coefficient of relative thermal activity of the layer materials [27]. Substituting the dependencies (23), (24) and (26), (27) to the transformed boundary conditions (19)–(22), the following system of four linear equations with respect to the function $A_{l}(p)$, $B_{l}(p)$, l=1,2 was obtained:

(29) $$A_{1}(p)+\varepsilon A_{2}(p)=-\frac{1}{p\sqrt{p}},\;A_{1}(p)-\varepsilon A_{2}(p)-\frac{Bi}{\sqrt{p}}[B_{1}(p)-B_{2}(p)]=0,$$

(30) $$b_{1}(p)A_{1}(p)+a_{1}(p)B_{1}(p)=0,\;b_{2}(p)A_{2}(p)+a_{2}(p)B_{2}(p)=0,$$

where

(31) $$a_{1}(p)=\mathrm{sh}(\sqrt{p})+\frac{Bi_{1}}{\sqrt{p}}\mathrm{ch}(\sqrt{p}),\;b_{1}(p)=\mathrm{ch}(\sqrt{p})+\frac{Bi_{1}}{\sqrt{p}}\mathrm{sh}(\sqrt{p}),$$

(32) $$a_{2}(p)=\delta \;\mathrm{sh}(\delta \sqrt{p}\;)+\frac{Bi_{2}}{\sqrt{p}}\mathrm{ch}(\delta \sqrt{p}\;),\;b_{2}(p)=\delta \;\mathrm{ch}(\delta \sqrt{p}\;)+\frac{Bi_{2}}{\sqrt{p}}\mathrm{sh}(\delta \sqrt{p}\;).$$

The solution of the system of Equations (29)–(32) was found in the form:

(33) $$A_{l}(p)=\;-\frac{\Delta_{A_{l}}(p)}{p\sqrt{p}\;\Psi (p)},\;B_{l}(p)=\frac{\Delta_{B_{l}}(p)}{p\sqrt{p}\;\Psi (p)},\;l=1,\;2,$$

where

(34) $$\Psi (p)=\Delta_{A_{1}}(p)+\varepsilon \Delta_{A_{2}}(p),$$

(35) $$\Delta_{A_{1}}(p)=a_{1}(p)c_{2}(p),\;\Delta_{B_{1}}(p)=b_{1}(p)c_{2}(p),$$

(36) $$\Delta_{A_{2}}(p)=a_{2}(p)c_{1}(p),\;\Delta_{B_{2}}(p)=b_{2}(p)c_{1}(p),$$

(37) $$c_{1}(p)=a_{1}(p)+\frac{Bi}{\sqrt{p}}b_{1}(p),\;b_{2}(p)=\delta \;\mathrm{ch}(\delta \sqrt{p}\;)+\frac{Bi_{2}}{\sqrt{p}}\mathrm{sh}(\delta \sqrt{p}\;).$$

After substituting the functions $A_{l}(p)$ and $B_{l}(p)$, l=1,2 (33)–(37) into Formulas (23), (24) and (26), (27), the following were obtained:

(38) $${\bar{\Theta }}^{*}(\zeta ,p)=\frac{\Phi_{1}(\zeta ,p)}{p\sqrt{p}\;\;\Psi (p)},\;\frac{d{\bar{\Theta }}^{*}(\zeta ,p)}{d\zeta }=\frac{Q_{1}(\zeta ,p)}{p\;\;\Psi (p)},\;0\leq \zeta \leq 1,$$

(39) $${\bar{\Theta }}^{*}(\zeta ,p)=\frac{\Phi_{2}(\zeta ,p)}{p\sqrt{p}\;\Psi (p)},\;K^{*}\frac{d{\bar{\Theta }}^{*}(\zeta ,p)}{d\zeta }=\varepsilon \frac{\;Q_{2}(\zeta ,p)}{p\;\;\Psi (p)},-d^{*}\leq \zeta \leq 0,$$

where

(40) $$\Phi_{1}(\zeta ,p)=-\Delta_{A_{1}}(p)\mathrm{sh}(\zeta \sqrt{p})+\Delta_{B_{1}}(p)\mathrm{ch}(\zeta \sqrt{p}),$$

(41) $$\Phi_{2}(\zeta ,p)=-\Delta_{A_{2}}(p)\mathrm{sh}\left(-\frac{\delta }{d^{*}}\zeta \sqrt{p}\right)+\Delta_{B_{2}}(p)\mathrm{ch}\left(-\frac{\delta }{d^{*}}\zeta \sqrt{p}\right),$$

(42) $$Q_{1}(\zeta ,p)=-\Delta_{A_{1}}(p)\mathrm{ch}(\zeta \sqrt{p})+\Delta_{B_{1}}(p)\mathrm{sh}(\zeta \sqrt{p}),$$

(43) $$Q_{2}(\zeta ,p)=\Delta_{A_{2}}(p)\mathrm{ch}\left(-\frac{\delta }{d^{*}}\zeta \sqrt{p}\right)-\Delta_{B_{2}}(p)\mathrm{sh}\left(-\frac{\delta }{d^{*}}\zeta \sqrt{p}\right).$$

The boundary values of the transformed solutions (38)–(43) were found in the form:

(44) $${\bar{\Theta }}^{*}(0^{+},p)=\frac{\Delta_{B_{1}}(p)}{p\sqrt{p}\;\Psi (p)},\;{\left.\frac{d{\bar{\Theta }}^{*}(\zeta ,p)}{d\zeta }\right|}_{\zeta =0^{+}}=-\frac{\Delta_{A_{1}}(p)}{p\;\Psi (p)},$$

(45) $${\bar{\Theta }}^{*}(0^{-},p)=\frac{\Delta_{B_{2}}(p)}{p\sqrt{p}\;\Psi (p)},\;K^{*}{\left.\frac{d{\bar{\Theta }}^{*}(\zeta ,p)}{d\zeta }\right|}_{\zeta =0^{-}}=\frac{\varepsilon \;\Delta_{A_{2}}(p)}{p\;\Psi (p)},$$

(46) $${\bar{\Theta }}^{*}(1,p)=\frac{\Phi_{1}(1,p)}{\;p\sqrt{p}\;\Psi (p)},\;{\left.\frac{d{\bar{\Theta }}^{*}(\zeta ,p)}{d\zeta }\right|}_{\zeta =1}=\frac{Q_{1}(1,p)}{p\;\;\Psi (p)},$$

(47) $${\bar{\Theta }}^{*}(-d^{*},p)=\frac{\Phi_{2}(-d^{*},p)}{\;p\sqrt{p}\;\Psi (p)},\;{\left.d^{*}\frac{d{\bar{\Theta }}^{*}(\zeta ,p)}{d\zeta }\right|}_{\zeta =-d^{*}}=\frac{\delta \;Q_{2}(-d^{*},p)}{p\;\;\Psi (p)},$$

where

(48) $$\Phi_{1}(1,p)=-\Delta_{A_{1}}(p)\mathrm{sh}(\sqrt{p})+\Delta_{B_{1}}(p)\mathrm{ch}(\sqrt{p}),$$

(49) $$Q_{1}(1,p)=-\Delta_{A_{1}}(p)\mathrm{ch}(\sqrt{p})+\Delta_{B_{1}}(p)\mathrm{sh}(\sqrt{p}),$$

(50) $$\Phi_{2}(-d^{*},p)=-\Delta_{A_{2}}(p)\mathrm{sh}(\delta \sqrt{p}\;)+\Delta_{B_{2}}(p)\mathrm{ch}(\delta \sqrt{p}\;),$$

(51) $$Q_{2}(-d^{*},p)=\Delta_{A_{2}}(p)\mathrm{ch}(\delta \sqrt{p}\;)-\Delta_{B_{2}}(p)\mathrm{sh}(\delta \sqrt{p}\;).$$

Taking into account the form of the functions Ψ(p), $\Delta_{A_{l}}(p)$, $\Delta_{B_{l}}(p)$, l=1,2 (34)–(37), from Formulas (44)–(51), it was established that:

(52) $${\left.K^{*}\frac{d{\bar{\Theta }}^{*}(\zeta ,p)}{d\zeta }\right|}_{\zeta =0^{-}}-\;{\left.\frac{d{\bar{\Theta }}^{*}(\zeta ,p)}{d\zeta }\right|}_{\zeta =0^{+}}=\frac{\varepsilon \;\Delta_{A_{2}}(p)+\Delta_{A_{1}}(p)}{p\;\Psi (p)}=\frac{\Psi (p)}{p\Psi (p)}=\frac{1}{p}$$

(53) $$\begin{array}{l} {\left.K^{*}\frac{d{\bar{\Theta }}^{*}(\zeta ,p)}{d\zeta }\right|}_{\zeta =0^{-}}+{\left.\frac{d{\bar{\Theta }}^{*}(\zeta ,p)}{d\zeta }\right|}_{\zeta =0^{+}}-Bi\;[{\bar{\Theta }}^{*}(0^{+},p)-{\bar{\Theta }}^{*}(0^{-},p)]=\\ \;=\frac{\varepsilon \;\Delta_{A_{2}}(p)-\Delta_{A_{1}}(p)}{p\;\Psi (p)}-\frac{Bi}{\sqrt{p}}\left[\frac{\Delta_{B_{1}}(p)-\Delta_{B_{2}}(p)}{p\;\Psi (p)}\right]=\frac{c_{2}(p)c_{1}(p)-c_{1}(p)c_{2}(p)}{p\;\Psi (p)}=0, \end{array}$$

(54) $$\begin{array}{l} {\left.\frac{d{\bar{\Theta }}^{*}(\zeta ,p)}{d\zeta }\right|}_{\zeta =1}+Bi_{1}\;{\bar{\Theta }}^{*}(1,p)=\\ =\frac{[-\Delta_{A_{1}}(p)\mathrm{sh}(\sqrt{p})+\Delta_{B_{1}}(p)\mathrm{ch}(\sqrt{p})]}{p\;\Psi (p)}+\frac{Bi_{1}}{\sqrt{p}}\left[\frac{-\Delta_{A_{1}}(p)\mathrm{sh}(\sqrt{p})+\Delta_{B_{1}}(p)\mathrm{ch}(\sqrt{p})}{p\;\Psi (p)}\right]=\\ =\frac{\left[-a_{1}(p)b_{1}(p)+a_{1}(p)b_{1}(p)]c_{2}(p\right)}{p\;\Psi (p)}=0, \end{array}$$

(55) $$\begin{array}{l} {\left.d^{*}\frac{d{\bar{\Theta }}^{*}(\zeta ,p)}{d\zeta }\right|}_{\zeta =-d^{*}}-Bi_{2}\;{\bar{\Theta }}^{*}(-d^{*},p)=\\ \;\;=\frac{\delta [\Delta_{A_{2}}(p)\mathrm{ch}(\delta \sqrt{p})-\Delta_{B_{2}}(p)\mathrm{sh}(\delta \sqrt{p})]}{p\;\Psi (p)}-\frac{Bi_{2}}{\sqrt{p}}\left[\frac{-\Delta_{A_{2}}(p)\mathrm{sh}(\delta \sqrt{p})+\Delta_{B_{2}}(p)\mathrm{ch}(\delta \sqrt{p})}{p\;\Psi (p)}\right]=\\ \;=\frac{\left[a_{2}(p)b_{2}(p)-a_{2}(p)b_{2}(p)]c_{1}(p\right)}{p\;\Psi (p)}=0. \end{array}$$

Dependencies (52)–(55) confirm the fact that Formulas (38) and (39) are solutions of the boundary-value problem (17)–(22) in the space of the Laplace integral transform. The performed verification confirms the correctness of the obtained solutions in the transform space. In the next section, the corresponding solution in the original space will be obtained.

## 4. Exact Solution in the Space of Originals

Considering the relations [26]:

(56) $$\mathrm{sh}(x)=-i\sin(ix),\;\mathrm{ch}(x)=\cos(ix),\;i\equiv \sqrt{-1},$$

in Formulas (31), (32), (37) and denoting:

(57) $$\mu =i\sqrt{p},$$

it was found that:

(58) $$a_{l}(p)=ia_{l}^{*}(\mu ),\;b_{l}(p)=b_{l}^{*}(\mu ),\;c_{l}(p)=ic_{l}^{*}(\mu ),\;l=1,\;2,$$

where

(59) $$a_{1}^{*}(\mu )=-\sin(\mu )+Bi_{1}\mu^{-1}\cos(\mu ),\;b_{1}^{*}(\mu )=\cos(\mu )+Bi_{1}\mu^{-1}\sin(\mu ),$$

(60) $$a_{2}^{*}(\mu )=-\delta \sin(\delta \mu )+Bi_{2}\mu^{-1}\cos(\delta \mu ),\;b_{2}^{*}(\mu )=\delta \cos(\delta \mu )+Bi_{2}\mu^{-1}\sin(\delta \mu ),$$

(61) $$c_{1}^{*}(\mu )=a_{1}^{*}(\mu )+Bi\mu^{-1}b_{1}^{*}(\mu ),\;c_{2}^{*}(\mu )=\varepsilon \;a_{2}^{*}(\mu )+Bi\mu^{-1}b_{2}^{*}(\mu ).$$

By substituting the functions $a_{l}(p)$, $b_{l}(p)$ and $c_{l}(p)$, l=1, 2 (58)–(61) into Formulas (34)–(36) and (40), (41), the following relations were obtained:

(62) $$\Delta_{A_{l}}(p)=-\Delta_{A_{l}}^{*}(\mu ),\;\Delta_{B_{l}}(p)=i\Delta_{B_{l}}^{*}(\mu ),\;\Phi_{l}(\zeta ,p)=i\Phi_{l}^{*}(\zeta ,\mu ),\;l=1,\;2,\;\Psi (p)=-\Psi^{*}(\mu ),$$

where

(63) $$\Phi_{1}^{*}(\zeta ,\mu )=-\Delta_{A_{1}}^{*}(\mu )\sin(\zeta \mu )+\Delta_{B_{1}}^{*}(\mu )\cos(\zeta \mu ),$$

(64) $$\Phi_{2}^{*}(\zeta ,\mu )=-\Delta_{A_{2}}^{*}(\mu )\sin\left(-\frac{\delta }{d^{*}}\zeta \mu \right)+\Delta_{B_{2}}^{*}(\mu )\cos\left(-\frac{\delta }{d^{*}}\zeta \mu \right),$$

(65) $$\Psi^{*}(\mu )=\Delta_{A_{1}}^{*}(\mu )+\varepsilon \;\Delta_{A_{2}}^{*}(\mu ),$$

(66) $$\Delta_{A_{1}}^{*}(\mu )=a_{1}^{*}(\mu )c_{2}^{*}(\mu ),\;\Delta_{B_{1}}^{*}(p)=b_{1}^{*}(\mu )c_{2}^{*}(\mu ),$$

(67) $$\Delta_{A_{2}}^{*}(\mu )=a_{2}^{*}(\mu )c_{1}^{*}(\mu ),\;\Delta_{B_{2}}^{*}(p)=b_{2}^{*}(\mu )c_{1}^{*}(\mu ).$$

After taking into account Formulas (62)–(67) in solutions (38) and (39), it was found:

(68) $${\bar{\Theta }}^{*}(\zeta ,p)=-\frac{\Phi_{1}^{*}(\zeta ,\mu )}{\mu^{3}\;\;\Psi^{*}(\mu )},\;0\leq \zeta \leq 1,\;{\bar{\Theta }}^{*}(\zeta ,p)=-\frac{\Phi_{2}^{*}(\zeta ,\mu )}{\mu^{3}\;\;\Psi^{*}(\mu )},\;-d^{*}\leq \zeta \leq 0.$$

The transition from the transforms (68) to the originals was carried out using the decomposition theorem [27]. On this basis, the dimensionless temperature rise was written in the form:

(69) $$\Theta^{*}(\zeta ,\tau )=\Theta_{1,0}^{*}(\zeta )+{\tilde{\Theta }}_{1}^{*}(\zeta ,\tau ),\;0\leq \zeta \leq 1,\;\;\tau \geq 0,$$

(70) $$\Theta^{*}(\zeta ,\tau )=\Theta_{2,0}^{*}(\zeta )+{\tilde{\Theta }}_{2}^{*}(\zeta ,\tau ),-d^{*}\leq \zeta \leq 0,\;\tau \geq 0,$$

where

(71) $$\Theta_{l,0}^{*}(\zeta )=\underset{\mu \to 0}{\lim}\frac{\Phi_{l}^{*}(\zeta ,\mu )}{\mu \;\Psi^{*}(\mu )},\;l=1,2,$$

(72) $${\tilde{\Theta }}_{l}^{*}(\zeta ,\tau )=2\sum_{n=1}^{\infty }\frac{\Phi_{l}^{*}(\zeta ,\mu_{n})}{\mu_{n}^{2}\;{\tilde{\Psi }}^{*}(\mu_{n})}\exp(-\mu_{n}^{2}\tau ),\;l=1,2,$$

$\mu_{n}>0$, n=1,2,…, are positive single roots of the characteristic equation:

(73) $$\Psi^{*}(\mu )=0,$$

and in Formulas (72), it was taken into account that $d\mu /dp=-0.5\mu^{-1}$. Using the functions $a_{l}^{*}(\mu )$, $b_{l}^{*}(\mu )$ and $c_{l}^{*}(\mu )$, l=1, 2 (59)–(61), from Formulas (65)–(67) it was found:

(74) $${\tilde{\Psi }}^{*}(\mu )\equiv d\Psi^{*}(\mu )/d\mu ={\tilde{a}}_{1}^{*}(\mu )c_{2}^{*}(\mu )+a_{1}^{*}(\mu ){\tilde{c}}_{2}^{*}(\mu )+\varepsilon [{\tilde{a}}_{2}^{*}(\mu )c_{1}^{*}(\mu )+a_{2}^{*}(\mu ){\tilde{c}}_{1}^{*}(\mu )],$$

where

(75) $${\tilde{a}}_{1}^{*}(\mu )=-b_{1}^{*}(\mu )-Bi_{1}\mu^{-2}\cos(\mu ),\;{\tilde{b}}_{1}^{*}(\mu )=a_{1}^{*}(\mu )-Bi_{1}\mu^{-2}\sin(\mu ),$$

(76) $${\tilde{a}}_{2}^{*}(\mu )=-\delta b_{2}^{*}(\mu )-Bi_{2}\mu^{-2}\cos(\delta \mu ),\;{\tilde{b}}_{2}^{*}(\mu )=\delta a_{2}^{*}(\mu )-Bi_{2}\mu^{-2}\sin(\delta \mu ),$$

(77) $${\tilde{c}}_{1}^{*}(\mu )={\tilde{a}}_{1}^{*}(\mu )+Bi\mu^{-1}[{\tilde{b}}_{1}^{*}(\mu )-\mu^{-1}b_{1}^{*}(\mu )],\;{\tilde{c}}_{2}^{*}(\mu )=\varepsilon \;{\tilde{a}}_{2}^{*}(\mu )+Bi\mu^{-1}[{\tilde{b}}_{2}^{*}(\mu )-\mu^{-1}b_{2}^{*}(\mu ).$$

The stationary components of the temperature rise $\Theta_{l,0}^{*}(\zeta )$, l=1, 2 (71) were sought, taking into account that at small argument values:

(78) sin(x)≅x, cos(x)≅1.

Then from Formulas (59)–(61) we found:

(79) $$a_{1}^{*}(\mu )≅-\mu +Bi_{1}\mu^{-1},\;b_{1}^{*}(\mu )≅1+Bi_{1},\;c_{1}^{*}(\mu )≅-\mu +\alpha_{1}\mu^{-1},$$

(80) $$a_{2}^{*}(\mu )≅-\delta^{2}\mu +Bi_{2}\mu^{-1},\;b_{2}^{*}(\mu )≅\delta (1+Bi_{2}),\;c_{2}^{*}(\mu )≅-\varepsilon \delta^{2}\mu +\alpha_{2}\mu^{-1},$$

where

(81) $$\alpha_{1}=Bi_{1}+Bi(1+Bi_{1}),\;\alpha_{2}=\varepsilon Bi_{2}+Bi\delta (1+Bi_{2}).$$

After substituting the relations (79)–(81) into Formulas (66) and (67), it was obtained:

(82) $$\Delta_{A_{1}}^{*}(\mu )≅-(1-Bi_{1}\mu^{-2})(\alpha_{2}-\varepsilon \delta^{2}\mu^{2}),\;\Delta_{B_{1}}^{*}(\mu )≅\mu^{-1}(1+Bi_{1})(\alpha_{2}-\varepsilon \delta^{2}\mu^{2}),$$

(83) $$\Delta_{A_{2}}^{*}(\mu )≅-(\delta^{2}-Bi_{2}\mu^{-2})(\alpha_{1}-\mu^{2}),\;\Delta_{B_{2}}^{*}(\mu )≅\mu^{-1}\delta (1+Bi_{2})(\alpha_{1}-\mu^{2}).$$

Next, after substituting the relations (82) and (83) into the Formulas (63)–(65), the following was established:

(84) $$\Phi_{1}^{*}(\zeta ,\mu )≅\mu^{-1}(\alpha_{2}-\varepsilon \delta^{2}\mu^{2})[1+Bi_{1}+(\mu^{2}-Bi_{1})\zeta ],$$

(85) $$\Phi_{2}^{*}(\zeta ,\mu )≅\mu^{-1}\delta (\alpha_{1}-\mu^{2})\left[1+Bi_{2}-(\delta^{2}\mu^{2}-Bi_{2})\frac{\zeta }{d^{*}}\right],$$

(86) $$\Psi^{*}(\mu )≅\mu^{-2}[(Bi_{1}-\mu^{2})(\alpha_{2}-\varepsilon \delta^{2}\mu^{2})+\varepsilon (Bi_{2}-\delta^{2}\mu^{2})(\alpha_{1}-\mu^{2})].$$

Substituting the function $\Phi_{l}^{*}(\zeta ,\mu )$, l=1, 2 (84), (85) and $\mu \Psi^{*}(\mu )$ (86) into the right-hand side of Formula (71) and passing to the limit μ→0, the stationary components of the temperature rise were found in the form:

(87) $$\Theta_{1,0}^{*}(\zeta )=\alpha^{-1}\phi_{1}(\zeta ),\;\;0\leq \zeta \leq 1,\;\Theta_{2,0}^{*}(\zeta )=\alpha^{-1}\phi_{2}(\zeta ),\;\;-d^{*}\leq \zeta \leq 0,$$

where

(88) $$\phi_{1}(\zeta )=\alpha_{2}[1+Bi_{1}(1-\zeta )],\;\phi_{2}(\zeta )=\alpha_{1}\delta \left[1+Bi_{2}\left(1+\frac{\zeta }{d^{*}}\right)\right],\;\alpha =\alpha_{2}Bi_{1}+\varepsilon \alpha_{1}Bi_{2},$$

and the coefficients $\alpha_{l}$, l=1,2 are defined by Formula (81):

Therefore, the exact solution of the thermal problem of friction (9)–(15) has the form (69), (70) with stationary $\Theta_{l,0}^{*}(\zeta )$ (87), (88) and transient ${\tilde{\Theta }}_{l}^{*}(\zeta ,\tau )$, l=1, 2 (72) components. We will prove that this solution satisfies the boundary conditions (11)–(14). For this purpose, differentiating Formulas (87) and (88) with respect to the variable ζ, the following form of the intensity of the heat flows was found:

(89) $$q_{1,0}^{*}\equiv -\frac{d\Theta_{1,0}^{*}(\zeta )}{d\zeta }=\frac{\alpha_{2}Bi_{1}}{\alpha },\;q_{2,0}^{*}\equiv K^{*}\frac{d\Theta_{2,0}^{*}(\zeta )}{d\zeta }=\frac{\varepsilon \alpha_{1}Bi_{2}}{\alpha },$$

from which it leads that:

(90) $$q_{1,0}^{*}+q_{2,0}^{*}=(\alpha_{2}Bi_{1}+\varepsilon \alpha_{1}Bi_{2})\alpha^{-1}=1,$$

(91) $$q_{2,0}^{*}-q_{1,0}^{*}=(\varepsilon \alpha_{1}Bi_{2}-\alpha_{2}Bi_{1})\alpha^{-1}.$$

On the other hand, Formulas (81), (87), and (88) were written as follows:

(92) $$\begin{array}{l} Bi[\Theta_{1,0}^{*}(0^{+})-\Theta_{2,0}^{*}(0^{-})]=Bi[\alpha_{2}(1+Bi_{1})-\alpha_{1}\delta (1+Bi_{2})]\alpha^{-1}=\\ =Bi[\alpha_{2}(\alpha_{1}-Bi_{1})Bi^{-1}-\alpha_{1}(\alpha_{2}-\varepsilon Bi_{2})Bi^{-1}]=(\varepsilon \alpha_{1}Bi_{2}-\alpha_{2}Bi_{1})\alpha^{-1}. \end{array}$$

The equalities (90)–(92) obtained in this way confirm that the components $\Theta_{l,0}^{*}(\zeta )$, l=1, 2 (87), (88) satisfy the boundary conditions (11) and (12). The fulfilment of the remaining two boundary conditions (13) and (14) is proved by the following equalities:

(93) $$q_{1,0}^{*}-Bi_{1}\Theta_{1,0}^{*}(1)=\alpha_{2}Bi_{1}\alpha^{-1}-Bi_{1}\alpha_{2}\alpha^{-1}=0,$$

(94) $$d^{*}{\left(K^{*}\right)}^{-1}q_{2,0}^{*}-Bi_{2}\Theta_{2,0}^{*}(-d^{*})=\delta \alpha_{1}Bi_{2}\alpha^{-1}-Bi_{2}\delta \alpha_{1}\alpha^{-1}=0.$$

It should be noted that the stationary components $\Theta_{l,0}^{*}(\zeta )$, l=1, 2 (87), (88) of the dimensionless temperature rise $\Theta^{*}(\zeta ,\tau )$ (69), (70) via equality (90) already satisfy the only inhomogeneous boundary condition (11). Therefore, it will be proven below that the non-stationary components ${\tilde{\Theta }}_{l}^{*}(\zeta ,\tau )$, l=1, 2 (72) satisfy only the corresponding homogeneous boundary conditions. Considering that on the right-hand side of Formula (72), only the function $\Phi_{l}^{*}(\zeta ,\mu )$, l=1, 2 contains the spatial variable ζ, it should be checked that:

(95) $${\left.K^{*}\frac{\partial \Phi_{2}^{*}(\zeta ,\mu )}{\partial \zeta }\right|}_{\zeta =0^{-}}-\;{\left.\frac{\partial \Phi_{1}^{*}(\zeta ,\mu )}{\partial \zeta }\right|}_{\zeta =0^{+}}=0,\;\tau \geq 0,$$

(96) $${\left.K^{*}\frac{\partial \Phi_{2}^{*}(\zeta ,\mu )}{\partial \zeta }\right|}_{\zeta =0^{-}}+\;{\left.\frac{\partial \Phi_{1}^{*}(\zeta ,\mu )}{\partial \zeta }\right|}_{\zeta =0^{+}}-Bi[\Phi_{1}^{*}(0^{+},\mu )-\Phi_{2}^{*}(0^{-},\mu )]=0,\;\tau \geq 0,$$

(97) $${\left.\frac{\partial \Phi_{1}^{*}(\zeta ,\mu )}{\partial \zeta }\right|}_{\zeta =1}+Bi_{1}\Phi_{1}^{*}(1,\mu )=0,\;\tau \geq 0,$$

(98) $$d^{*}{\left.\frac{\partial \Phi_{2}^{*}(\zeta ,\mu )}{\partial \zeta }\right|}_{\zeta =-d^{*}}-Bi_{2}\Phi_{2}^{*}(-d^{*},\mu )=0.$$

Taking into account Formulas (63), (64) and (66), (67), the partial derivatives were found as:

(99) $$\frac{\partial \Phi_{1}^{*}(\zeta ,\tau )}{\partial \zeta }=-\mu c_{2}^{*}(\mu )[a_{1}^{*}(\mu )\cos(\zeta \mu )+b_{1}^{*}(\mu )\sin(\zeta \mu )],$$

(100) $$\frac{\partial \Phi_{2}^{*}(\zeta ,\tau )}{\partial \zeta }=\frac{\delta }{d^{*}}\mu c_{1}^{*}(\mu )\left[a_{2}^{*}(\mu )\cos\left(-\frac{\delta }{d^{*}}\zeta \mu \right)+b_{2}^{*}(\mu )\sin\left(-\frac{\delta }{d^{*}}\zeta \mu \right)\right].$$

At $\zeta =0^{\pm }$ the derivatives (99) and (100) are equal:

(101) $${\left.\frac{\partial \Phi_{1}^{*}(\zeta ,\tau )}{\partial \zeta }\right|}_{\zeta =0^{+}}=-\mu a_{1}^{*}(\mu )c_{2}^{*}(\mu ),\;{\left.K^{*}\frac{\partial \Phi_{2}^{*}(\zeta ,\tau )}{\partial \zeta }\right|}_{\zeta =0^{-}}=\varepsilon \mu a_{2}^{*}(\mu )c_{1}^{*}(\mu ).$$

After substituting the Formulas (101) into the left side of equality (95) and taking into account the form (65)–(67) of the function $\Psi^{*}(\mu )$ and the characteristic Equation (73), it was obtained:

(102) $$\mu [a_{1}^{*}(\mu )c_{2}^{*}(\mu )+\varepsilon a_{2}^{*}(\mu )c_{1}^{*}(\mu )]=\mu \Psi^{*}(\mu )=0,\;\tau \geq 0.$$

Taking into account the derivatives (101) and Formulas (61), (63), (64), (66), (67) in the left side of equality (96), the following was determined:

(103) $$\begin{array}{l} \mu [\varepsilon a_{2}^{*}(\mu )c_{1}^{*}(\mu )-a_{1}^{*}(\mu )c_{2}^{*}(\mu )]-Bi[b_{1}^{*}(\mu )c_{2}^{*}(\mu )-b_{2}^{*}(\mu )c_{1}^{*}(\mu )]=\\ =\mu \{c_{1}^{*}(\mu )[\varepsilon a_{2}^{*}(\mu )+Bi\mu^{-1}b_{2}^{*}(\mu )]-c_{2}^{*}(\mu )[a_{1}^{*}(\mu )+Bi\mu^{-1}b_{1}^{*}(\mu )]\}=\\ =\;\mu [c_{1}^{*}(\mu )c_{2}^{*}(\mu )-c_{2}^{*}(\mu )c_{1}^{*}(\mu )]=0. \end{array}$$

Considering the values of the function $\Phi_{1}^{*}(\zeta ,\tau )$ (63), (66), its derivative (99) at ζ=1 and the relations (59) on the left side of the equality (97), we obtain:

(104) $$\begin{array}{l} -\mu c_{2}^{*}(\mu )[a_{1}^{*}(\mu )\cos(\mu )+b_{1}^{*}(\mu )\sin(\mu )]-Bi_{1}c_{2}^{*}(\mu )[a_{1}^{*}(\mu )\sin(\mu )-b_{1}^{*}(\mu )\cos(\mu )]=\\ =-\mu c_{2}^{*}(\mu )\{a_{1}^{*}(\mu )[\cos(\mu )+Bi_{1}\mu^{-1}\sin(\mu )]+b_{1}^{*}(\mu )[\sin(\mu )-Bi_{1}\mu^{-1}\cos(\mu )]\}=\\ =-\mu c_{2}^{*}(\mu )[a_{1}^{*}(\mu )b_{1}^{*}(\mu )-b_{1}^{*}(\mu )a_{1}^{*}(\mu )]=0. \end{array}$$

Finally, based on Formulas (64), (67), (100) at $\zeta =-d^{*}$ and relations (60), the left side of equality (98) was written in the form:

(105) $$\begin{array}{l} \mu \delta c_{1}^{*}(\mu )[a_{2}^{*}(\mu )\cos(\delta \mu )+b_{2}^{*}(\mu )\sin(\delta \mu )]-Bi_{2}c_{1}^{*}(\mu )[-a_{2}^{*}(\mu )\sin(\delta \mu )+b_{2}^{*}(\mu )\cos(\delta \mu )]=\\ =\mu c_{1}^{*}(\mu )\{a_{2}^{*}(\mu )[\delta \cos(\delta \mu )+Bi_{2}\mu^{-1}\sin(\delta \mu )]+b_{2}^{*}(\mu )[\delta \sin(\delta \mu )-Bi_{2}\mu^{-1}\cos(\delta \mu )]\}=\\ =\mu c_{1}^{*}(\mu )[a_{2}^{*}(\mu )b_{2}^{*}(\mu )-b_{2}^{*}(\mu )a_{2}^{*}(\mu )]=0. \end{array}$$

Thus, it has been proven that the solutions (69) and (70) satisfy the boundary conditions (11)–(14). The satisfaction of the initial condition (15) is carried out numerically by checking the fulfillment of the equation $\Theta_{l,0}^{*}(\zeta )+\Theta_{l}^{*}(\zeta ,0)=0$, $-d^{*}\leq \zeta \leq 1$, l=1, 2.

## 5. Some Specific Cases

On the basis of the solution presented above, some specific cases will be considered, which are important from the application point of view.

### 5.1. Maximum Temperature

The maximum temperature is reached on the friction surfaces of the layers. From Formulas (88) and (89), it follows that the stationary components of the dimensionless rise in the maximum temperature are equal to:

(106) $$\Theta_{1,0}^{*}\equiv \Theta_{1,0}^{*}(0^{+})=\alpha^{-1}\phi_{1,0},\;\Theta_{2,0}^{*}\equiv \Theta_{2,0}^{*}(0^{-})=\alpha^{-1}\phi_{2,0},$$

where

(107) $$\phi_{1,0}\equiv \phi_{1}(0)=\alpha_{2}(1+Bi_{1}),\;\phi_{2,0}\equiv \phi_{2}(0)=\delta \alpha_{1}(1+Bi_{2}),$$

and the coefficients $\alpha_{l},\;\;l=1,\;2$ and α were calculated from Formulas (81) and (88), respectively.Then, by putting $\zeta =0^{\pm }$ in Formulas (63), (64) and (72), the non-stationary components of the maximum temperature rise were written in the form:

(108) $${\tilde{\Theta }}_{l}^{*}(\tau )=2\sum_{n=1}^{\infty }\frac{\Phi_{l}^{*}(\mu_{n})}{\mu_{n}^{2}\;{\tilde{\Psi }}^{*}(\mu_{n})}\exp(-\mu_{n}^{2}\tau ),\;\tau \geq 0,\;\;l=1,\;2,$$

where

(109) $$\Phi_{1}^{*}(\mu )=b_{1}^{*}(\mu )c_{2}^{*}(\mu ),\;\Phi_{2}^{*}(\mu )=b_{2}^{*}(\mu )c_{1}^{*}(\mu ),$$

and the roots $\mu_{n}>0$, n=1,2,…, were determined from the characteristic Equation (73) with the function $\Psi^{*}(\mu )$ (65)–(67) and its derivative ${\tilde{\Psi }}^{*}(\mu )$ (74)–(77). Therefore, the dimensionless temperature rise in the friction layer surfaces $\zeta =0^{\pm }$ were determined from the Formulas

(110) $$\Theta_{l}^{*}(\tau )=\Theta_{l,0}^{*}+{\tilde{\Theta }}_{l}^{*}(\tau ),\;\;\tau \geq 0,\;l=1,2,$$

where the components $\Theta_{l,0}^{*}$ and ${\tilde{\Theta }}_{l}^{*}(\tau )$ were calculated using Formulas (106), (107) and (108), (109), respectively.

### 5.2. Heat Flux Intensity

Additional important application parameters are the dimensionless intensities of heat fluxes [28], defined by the Fourier’s law [29]:

(111) $$q^{*}(\zeta ,\tau )\equiv \frac{q(z,t)}{q_{0}}=\left\{\begin{array}{l} -\frac{\partial \Theta^{*}(\zeta ,\tau )}{\partial \zeta },\;\;0\leq \zeta \leq 1,\;\;\tau \geq 0,\\ K^{*}\frac{\partial \Theta^{*}(\zeta ,\tau )}{\partial \zeta },\;-d^{*}\leq \zeta \leq 0,\;\tau \geq 0, \end{array}\right.$$

With consideration of the solution (69), (70), the heat flux intensities were written in the form

(112) $$q^{*}(\zeta ,\tau )=\left\{\begin{array}{l} q_{1,0}^{*}+{\tilde{q}}_{1}^{*}(\zeta ,\tau ),\;\;0\leq \zeta \leq 1,\;\;\tau \geq 0,\\ q_{2,0}^{*}+{\tilde{q}}_{2}^{*}(\zeta ,\tau ),\;\;-d^{*}\leq \zeta \leq 0,\;\;\tau \geq 0, \end{array}\right.$$

where the stationary components $q_{l,0}^{*}$, l=1, 2 were given by the Formula (89). Taking into account the form of solutions (72) and partial derivatives (99) and (100), the non-stationary components ${\tilde{q}}_{l}^{*}(\zeta ,\tau )$, l=1, 2 in Formula (112) were obtained:

(113) $${\tilde{q}}_{1}^{*}(\zeta ,\tau )=2\sum_{n=1}^{\infty }\frac{Q_{1}^{*}(\zeta ,\mu_{n})}{\mu_{n}\;{\tilde{\Psi }}^{*}(\mu_{n})}\exp(-\mu_{n}^{2}\tau ),\;0\leq \zeta \leq 1,\;\tau \geq 0,$$

(114) $${\tilde{q}}_{2}^{*}(\zeta ,\tau )=2\sum_{n=1}^{\infty }\frac{Q_{2}^{*}(\zeta ,\mu_{n})}{\mu_{n}\;{\tilde{\Psi }}^{*}(\mu_{n})}\exp(-\mu_{n}^{2}\tau ),\;\;-d^{*}\leq \zeta \leq 0,\;\tau \geq 0,$$

where

(115) $$Q_{1}^{*}(\zeta ,\mu )=c_{2}^{*}(\mu )[a_{1}^{*}(\mu )\cos(\zeta \mu )+b_{1}^{*}(\mu )\sin(\zeta \mu )],$$

(116) $$Q_{2}^{*}(\zeta ,\mu )=\varepsilon c_{1}^{*}(\mu )\left[a_{2}^{*}(\mu )\cos\left(-\frac{\delta }{d^{*}}\zeta \mu \right)+b_{2}^{*}(\mu )\sin\left(-\frac{\delta }{d^{*}}\zeta \mu \right)\right].$$

From Formulas (113)–(116), the non-stationary components of the heat flux intensity at $\zeta =0^{\pm }$ were determined as:

(117) $${\tilde{q}}_{l}^{*}(\tau )=2\sum_{n=1}^{\infty }\frac{Q_{l}^{*}(\mu_{n})}{\mu_{n}\;{\tilde{\Psi }}^{*}(\mu_{n})}\exp(-\mu_{n}^{2}\tau ),\;\;\tau \geq 0,\;l=1,2,$$

where

(118) $$Q_{1}^{*}(\mu )=a_{1}^{*}(\mu )c_{2}^{*}(\mu ),\;Q_{2}^{*}(\mu )=a_{2}^{*}(\mu )c_{1}^{*}(\mu ).$$

Based on Formulas (112), (117) and (118) of the heat flux intensities on the friction surfaces $\zeta =0^{\pm }$ of the layers was presented in the form:

(119) $$q_{l}^{*}(\tau )=q_{l,0}^{*}+{\tilde{q}}_{l}^{*}(\tau ),\;\;\;\tau \geq 0,\;l=1,2.$$

The thermal resistance at the contact interface affects the redistribution of the heat flux contribution through the parameter h (the dimensionless Biot number Bi), which is inversely proportional to the thermal resistance. It appears in the expressions for the coefficients $\alpha_{1}$ and $\alpha_{2}$ (81) in the steady-state part of the solution, as well as in the coefficients $c_{1}^{*}$ and $c_{2}^{*}$ (61) in the transient part of the solution.

### 5.3. Perfect Thermal Contact of Friction

Perfect (full) friction thermal contact is realized when the sliding surfaces are so smooth that the thermal resistance of the contact surfaces can be neglected. In the mathematical model under consideration, this means that the coefficient of the thermal contact conductance h→∞ (Bi→∞). Then, as it results from boundary conditions (4) or (12), the temperature of the surfaces of the friction layer should be equal. Passing in Formulas (106), (107) to the limit Bi→∞, it follows that:

(120) $$\Theta_{0}^{*}\equiv \Theta_{1,0}^{*}=\Theta_{2,0}^{*}=\alpha^{-1}\phi_{0},$$

where

(121) $$\phi_{0}\equiv \phi_{1,0}=\phi_{2,0}=\delta (1+Bi_{1})(1+Bi_{2}),\;\alpha =\delta Bi_{1}(1+Bi_{2})+\varepsilon Bi_{2}(1+Bi_{1}).$$

Next, the solutions (108) and (109) at Bi→∞ were written in the form:

(122) $${\tilde{\Theta }}^{*}(\tau )\equiv {\tilde{\Theta }}_{1}^{*}(\tau )={\tilde{\Theta }}_{2}^{*}(\tau )=2\sum_{n=1}^{\infty }\frac{\Phi^{*}(\mu_{n})}{\mu_{n}^{2}\;{\tilde{\Psi }}^{*}(\mu_{n})}\exp(-\mu_{n}^{2}\tau ),\;\tau \geq 0,$$

where

(123) $$\Phi^{*}(\mu )\equiv \Phi_{1}^{*}(\mu )=\Phi_{2}^{*}(\mu )=b_{1}^{*}(\mu )b_{2}^{*}(\mu ),$$

(124) $${\tilde{\Psi }}^{*}(\mu )={\tilde{a}}_{1}^{*}(\mu )b_{2}^{*}(\mu )+a_{1}^{*}(\mu ){\tilde{b}}_{2}^{*}(\mu )+\varepsilon [{\tilde{a}}_{2}^{*}(\mu )b_{1}^{*}(\mu )+a_{2}^{*}(\mu ){\tilde{b}}_{2}^{*}(\mu )],$$

$\mu_{n}>0$, n=1,2,3,…, are the roots of the following equation:

(125) $$\Psi^{*}(\mu )=a_{1}^{*}(\mu )b_{2}^{*}(\mu )+\varepsilon a_{2}^{*}(\mu )b_{1}^{*}(\mu )=0,$$

and the function $a_{l}^{*}(\mu )$, $b_{l}^{*}(\mu )$ as well as their derivatives ${\tilde{a}}_{l}^{*}(\mu )$, ${\tilde{b}}_{l}^{*}(\mu )$, l=1,2 were determined from Formulas (59), (60) and (75), (76), respectively. Therefore, the dimensionless temperature rise for perfect friction thermal contact should be calculated as the sum:

(126) $$\Theta^{*}(\tau )\equiv \Theta_{0}^{*}+{\tilde{\Theta }}^{*}(\tau ),\;\tau \geq 0,$$

with components determined from Formulas (120)–(125).

### 5.4. Asymptotic Solution at Small Values of the Fourier Number

The asymptotic solution at small values of the Fourier number 0≤τ<<1 will be obtained based on the analysis of solutions (38) and (39) for large values of the Laplace integral transform parameter p (16). After taking into account the behaviour of hyperbolic functions at large values of the argument:

(127) sh(x)=ch(x)≅0.5exp(x),

from Formulas (31)–(37) and (40), (41), the following was found:

(128) $$\Phi_{1}(\zeta ,p)=a_{1}(p)a_{2}(p)\left(\varepsilon +\frac{Bi}{\sqrt{p}}\right)\exp(-\zeta \sqrt{p}),\;0\leq \zeta \leq 1,$$

(129) $$\Phi_{2}(\zeta ,p)=a_{1}(p)a_{2}(p)\left(1+\frac{Bi}{\sqrt{p}}\right)\exp\left(\frac{\delta }{d^{*}}\zeta \sqrt{p}\right),-d^{*}\leq \zeta \leq 0,$$

(130) $$\Psi (p)=a_{1}(p)a_{2}(p)\left[2\varepsilon +(1+\varepsilon )\frac{Bi}{\sqrt{p}}\right],$$

(131) $$a_{1}(p)=\frac{1}{2}\left(1+\frac{Bi_{1}}{\sqrt{p}}\right)\exp(\sqrt{p}),\;a_{2}(p)=\frac{1}{2}\left(\varepsilon +\frac{Bi_{2}}{\sqrt{p}}\right)\exp(\delta \sqrt{p}).$$

Taking into account the asymptotes (128)–(131), the transformed solutions (38) and (39) were written in the form:

(132) $${\bar{\Theta }}^{*}(\zeta ,p)=\frac{\exp(-\zeta \sqrt{p})}{2\varepsilon }\left[\frac{\;\varepsilon }{p(\sqrt{p}+\kappa )}+\frac{Bi\;}{p\sqrt{p}(\sqrt{p}+\kappa )}\right],\;\;0\leq \zeta \leq 1,$$

(133) $${\bar{\Theta }}^{*}(\zeta ,p)=\frac{\exp\left(\frac{\delta }{d^{*}}\zeta \sqrt{p}\right)}{2\varepsilon }\left[\frac{\;1}{p(\sqrt{p}+\kappa )}+\frac{Bi\;}{p\sqrt{p}(\sqrt{p}+\kappa )}\right],\;\;-d^{*}\leq \zeta \leq 0,$$

where

(134) $$\kappa =\frac{\left(1+\varepsilon \right)}{2\varepsilon }Bi.$$

Using the relations [30]:

(135) $$L^{-1}\left[\frac{\kappa \exp(-a\sqrt{p})}{p(\sqrt{p}+\kappa )};\;\tau \right]=\mathrm{erfc}\left(\frac{a}{2\sqrt{\tau }}\right)-\exp(\kappa^{2}\tau +\kappa a)\mathrm{erfc}\left(\frac{a}{2\sqrt{\tau }}+\kappa \sqrt{\tau }\right),\;a\geq 0,$$

(136) $$\begin{array}{l} L^{-1}\left[\frac{\kappa \exp(-a\sqrt{p})}{p\sqrt{p}(\sqrt{p}+\kappa )};\;\tau \right]=\\ =2\sqrt{\tau }\;\mathrm{ierfc}\;\left(\frac{a}{2\sqrt{\tau }}\right)-\frac{1}{\kappa }\left[\mathrm{erfc}\left(\frac{a}{2\sqrt{\tau }}\right)-\exp(\kappa^{2}\tau +\kappa a)\mathrm{erfc}\left(\frac{a}{2\sqrt{\tau }}+\kappa \sqrt{\tau }\right)\right], \end{array}$$

($\mathrm{ierfc}(x)=\pi^{-\frac{1}{2}}\exp(-x^{2})-x\;\mathrm{erfc}(x)$, erfc(x)=1−erf(x), erf(x) is the Gaussian error function [26]), the following asymptotic solutions for estimating the dimensionless temperature increases in the layers at the initial (0≤τ<<1) moments of the friction heating process were obtained from Formulas (132) and (133):

(137) $${\hat{\Theta }}^{*}(\zeta ,\tau )=\frac{2\sqrt{\tau }}{\left(1+\varepsilon \right)}\;\mathrm{ierfc}\;\left(\frac{\zeta }{2\sqrt{\tau }}\right)-\frac{\lambda }{2\kappa }\left[\mathrm{erfc}\left(\frac{\zeta }{2\sqrt{\tau }}\right)-\exp(\kappa^{2}\tau +\kappa \zeta )\mathrm{erfc}\left(\frac{\zeta }{2\sqrt{\tau }}+\kappa \sqrt{\tau }\right)\right],\;0\leq \zeta \leq 1,$$

(138) $$\begin{array}{l} {\hat{\Theta }}^{*}(\zeta ,\tau )=\frac{2\sqrt{\tau }}{\left(1+\varepsilon \right)}\;\mathrm{ierfc}\;\left(-\frac{\delta \zeta }{2d^{*}\sqrt{\tau }}\right)+\\ +\frac{\lambda }{2\kappa \varepsilon }\left[\mathrm{erfc}\;\left(-\frac{\delta \zeta }{2d^{*}\sqrt{\tau }}\right)-\exp(\kappa^{2}\tau -\kappa \frac{\delta \zeta }{d^{*}})\mathrm{erfc}\left(-\frac{\delta \zeta }{2d^{*}\sqrt{\tau }}+\kappa \sqrt{\tau }\right)\right],\;-d^{*}\leq \zeta \leq 0, \end{array}$$

where

(139) $$\lambda =\frac{1-\varepsilon }{1+\varepsilon }.$$

On the surface of friction $\zeta =0^{\pm }$ with Formulas (137) and (138), the following were found:

(140) $${\hat{\Theta }}_{1}^{*}(\tau )=\frac{2}{\left(1+\varepsilon \right)}\sqrt{\frac{\tau }{\pi }}-\frac{\lambda }{2\kappa }\left[1-\exp(\kappa^{2}\tau )\mathrm{erfc}(\kappa \sqrt{\tau })\right],\;{\hat{\Theta }}_{2}^{*}(\tau )=\frac{2}{\left(1+\varepsilon \right)}\sqrt{\frac{\tau }{\pi }}+\frac{\lambda }{2\kappa \varepsilon }\left[1-\exp(\kappa^{2}\tau )\mathrm{erfc}(\kappa \sqrt{\tau })\right],\;0\leq \tau <<1.$$

It should be noted that the solutions (137)–(140) do not include the Biot numbers $Bi_{l}$, l=1,2, or the relative thickness of layers $d^{*}$, and they coincide with the known asymptotic solutions for two homogeneous half-spaces ζ≥0 and ζ≤0 [31], respectively. Additionally, passing in Formulas (137)–(140) to the limit Bi→∞ (κ→∞), the dimensionless temperature rises at perfect thermal contact of friction of the layers in the initial heating period, were found in the known form [32]:

(141) $${\hat{\Theta }}^{*}(\zeta ,\tau )=\frac{2\sqrt{\tau }}{\left(1+\varepsilon \right)}\;\mathrm{ierfc}\;\left(\frac{\zeta }{2\sqrt{\tau }}\right),\;\;0\leq \zeta \leq 1,\;{\hat{\Theta }}^{*}(\zeta ,\tau )=\frac{2\sqrt{\tau }}{\left(1+\varepsilon \right)}\;\mathrm{ierfc}\;\left(-\frac{\delta \zeta }{2d^{*}\sqrt{\tau }}\right),\;\;-d^{*}\leq \zeta \leq 0,$$

(142) $${\hat{\Theta }}^{*}(\tau )\equiv {\hat{\Theta }}_{1}^{*}(\tau )={\hat{\Theta }}_{2}^{*}(\tau )=\frac{2}{\left(1+\varepsilon \right)}\sqrt{\frac{\tau }{\pi }},\;0\leq \tau <<1.$$

## 6. Numerical Analysis

The calculations were carried out for a friction system consisting of two layers, one of which was made of cermet FMC-11 ($K_{1}=35.005\;{\mathrm{Wm}}^{-1}K^{-1}$, $\;k_{1}=15.5\cdot 10^{-6}\;m^{2}s^{-1}$) and the other of grey cast iron ChNMKh ($K_{2}=52.17\;{\mathrm{Wm}}^{-1}K^{-1}$, $k_{2}=16.5\cdot 10^{-6}\;m^{2}s^{-1}$) [33]. These materials are commonly used in braking systems operating at volume and surface temperatures ≈973 K and ≈1273 K, respectively [34]. For the friction pair selected in this way, the following constant values of dimensionless parameters were determined from Formulas (8) and (28): $K^{*}=1.49$, $k^{*}=1.063$, $\varepsilon^{*}=1.446$. The input parameters defined by Formulas (8) are: the spatial variable ζ, the Fourier number τ, the Biot numbers Bi and $Bi_{l}$, l=1,2, the relative thickness of layers $d^{*}$. The influence of these parameters on the evolution of the dimensionless temperature increase $\Theta_{l,0}^{*}$ (106), (107), $\Theta_{l}^{*}(\tau )$ (110), ${\hat{\Theta }}_{l}^{*}(\tau )$ (140) and heat flux intensities $q_{l}^{*}(\tau )$ (119) on the friction surfaces $\zeta =0^{+}$ (l=1) and $\zeta =0^{-}$ (l=2) was investigated. It should be noted that when performing the numerical analysis in dimensionless form, there is no need to specify the value of the specific friction power q0. This parameter is used only to determine the temperature scaling factor $\Theta_{0}$.

The positive roots $\mu_{n}$, n=1,2,… of the characteristic Equation (73) were calculated by means of the bisection method, implemented in the RTBIS program [35]. In order to achieve the desired relative accuracy $10^{-4}$ in calculations for different values of operating parameters, up to 30 roots of the characteristic Equation (73) had to be considered.

**Figure 2:**
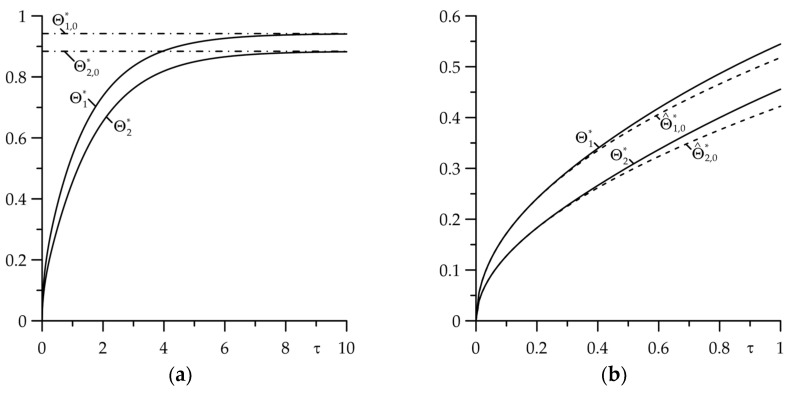
Evolutions of dimensionless temperature rise in the friction surfaces: (**a**) $\Theta_{l}^{*}(\tau )$ (continuous lines) and $\Theta_{l,0}^{*}$ (dash-dot lines), l=1,2; (**b**) $\Theta_{l}^{*}(\tau )$ (continuous lines) and ${\hat{\Theta }}_{l}^{*}(\tau )$ (dashed lines), l=1,2 for $Bi=1,\;\;Bi_{1}=Bi_{2}=100,\;\;d^{*}=1$.

The results of calculations presented in Figure 2 refer to the case of imperfect thermal contact of friction (Bi=1) of two layers of the same thickness ($d^{*}=1$) with the same high intensity of convective cooling on their free surfaces $\left(Bi_{1}=Bi_{2}=100\right)$. Consideration of the thermal contact conductance results in a temperature jump on the friction surfaces, while convective heat exchange with the surrounding environment on the free surfaces of the layers causes the temperature to reach a steady state after a certain period of the friction heating process (Figure 2a). For the considered case, the temperature estimation on the friction surfaces of the layers can be performed using the asymptotic solution (140) with high accuracy in the range of the Fourier number values 0≤τ≤0.5.

**Figure 3:**
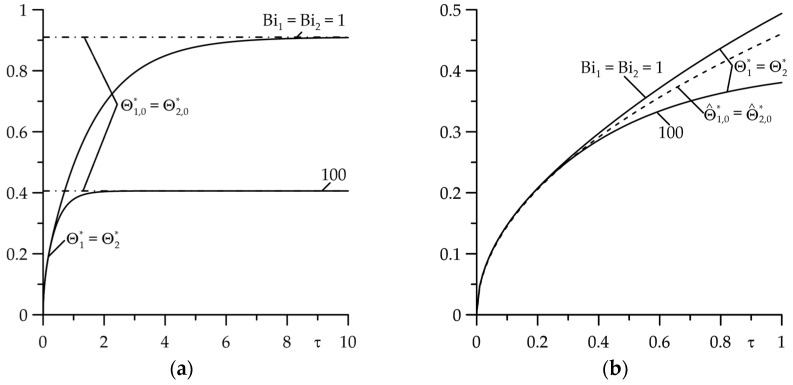
Evolutions of dimensionless temperature rise in the friction surfaces: (**a**) $\Theta_{l}^{*}(\tau )$ (continuous lines) and $\Theta_{l,0}^{*}$ (dash-dot lines), l=1,2; (**b**) $\Theta_{l}^{*}(\tau )$ (continuous lines) and ${\hat{\Theta }}_{l}^{*}(\tau )$ (dashed lines), l=1,2 at $Bi=100,\;\;d^{*}=1$ for two values of the Biot numbers $\;Bi_{1}=Bi_{2}=1$ and $\;Bi_{1}=Bi_{2}=100$.

Increasing the thermal contact conductance (reducing the thermal resistance) equalizes the temperature of the friction layers and at Bi=100 becomes practically the same on both surfaces (Figure 3). The so-called conditions of perfect thermal contact of friction then occur [36]. Increasing the intensity of convective cooling results in a significant temperature reduction, while simultaneously shortening the time to reach the stationary temperature (Figure 3a). In the initial moments of heating (with Fourier number values in the range 0≤τ≤0.4), the influence of convective cooling intensity on the temperature is negligible, and asymptotic solutions (140) can be used to determine it (Figure 3b).

**Figure 4:**
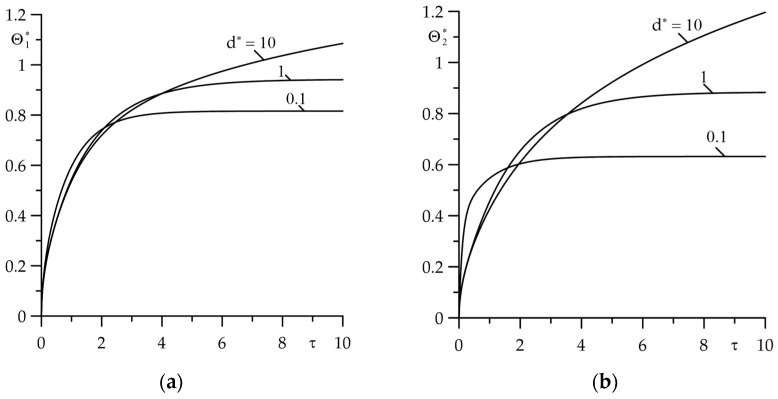
Dimensionless temperature variations on the friction surfaces: (**a**) $\Theta_{1}^{*}(\tau )$; (**b**) $\Theta_{2}^{*}(\tau )$ for three values of the relative layers thickness $d^{*}=0.1,\;1,\;10$ at $Bi=\;Bi_{1}=Bi_{2}=1$.

The effect of the relative layers thickness $d^{*}$ on the temperature of the friction surfaces of the layers is presented in Figure 4. It was found that in the considered range of the Fourier number 0≤τ≤10, the steady state temperature is achieved for two values (0.1 and 1) of the parameter $d^{*}$. However, at $d^{*}=10$, the temperature increases monotonically with the increase in the Fourier number τ. Such a time profile of temperature is characteristic of friction heating of two semi-spaces [32].

**Figure 5:**
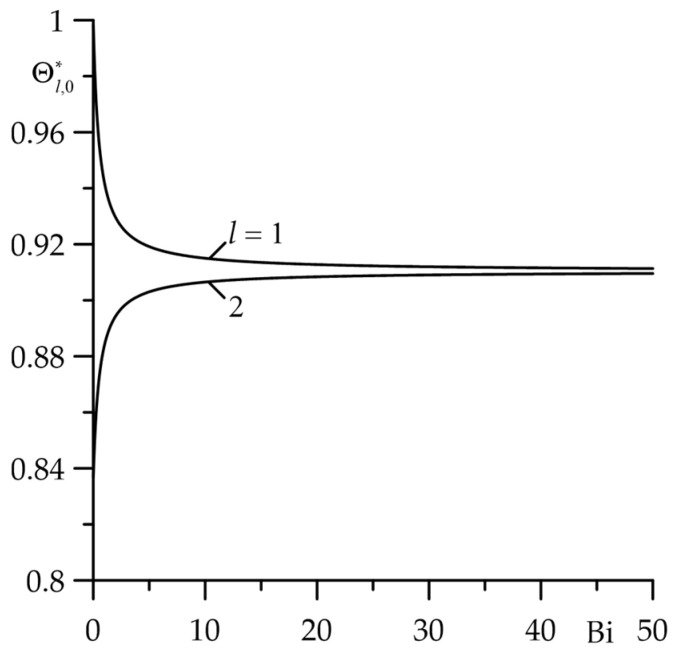
Dependence of the dimensionless rise in the stationary temperature $\Theta_{l,0}^{*}$, l=1,2 of the friction surfaces on the Biot number Bi at $\;Bi_{1}=Bi_{2}=d^{*}=1$.

**Figure 6:**
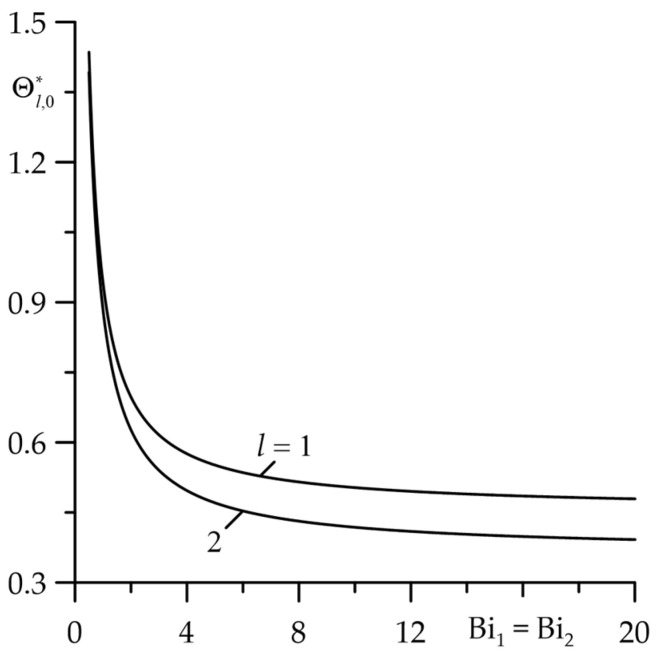
Influence of Biot numbers $Bi_{1}=Bi_{2}$ on dimensionless increases in stationary temperature $\Theta_{l,0}^{*}$, l=1,2 of friction surfaces at $\;Bi=d^{*}=1$.

**Figure 7:**
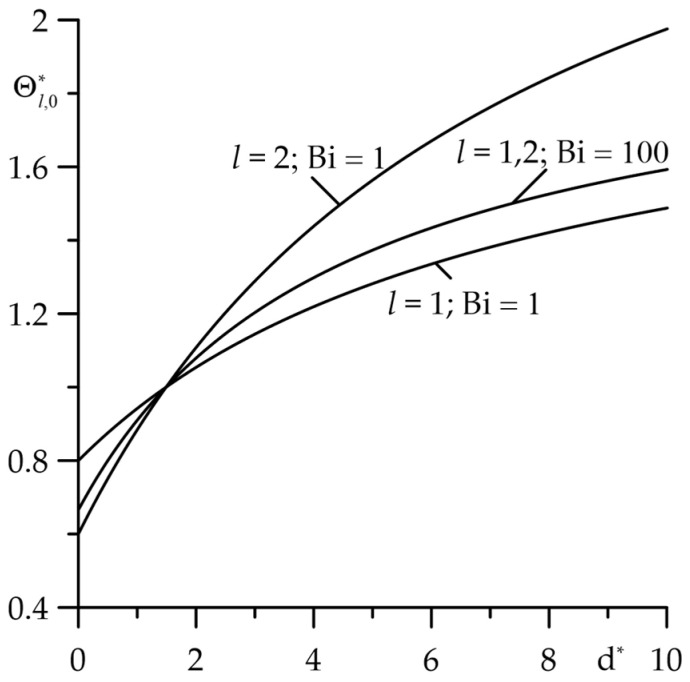
Dependence of the dimensionless increase in the stationary temperature $\Theta_{l,0}^{*}$, l=1,2 of the friction surfaces on the relative thickness $d^{*}$ of the layers for two values of the Biot number Bi=1 and Bi=100 at $\;Bi_{1}=Bi_{2}=1$.

As shown in Figure 2, Figure 3 and Figure 4, with appropriate selection of the input parameters, after some time from the start of friction heating, the temperature reaches its maximum value and then remains practically unchanged. This is then referred to as a steady-state temperature condition. The effect of input parameters on the dimensionless increase in the stationary temperature $\Theta_{l,0}^{*}$ (106), (107) is presented in Figure 5, Figure 6 and Figure 7. First, the effect of thermal contact conductance h (the Biot number Bi) on the temperature (dimensionless temperature increase) of the friction surface of layers $\zeta =0^{\pm }$ was investigated (Figure 5). The maximum temperature jump of these surfaces occurs at Bi→0, when their thermal contact resistance is the greatest. Increasing Bi parameter causes the temperature of the friction surface layers to equalize. In the case under consideration, the thermal contact of the friction layers can be assumed to be perfect at Bi≥50, and the steady-state temperature can be determined using solutions given by Formulas (120) and (121).

The attempt to thermally isolate the friction surfaces ($Bi_{l}\to 0$,l=1,2) causes a rapid rise in temperature while simultaneously equalizing it on both surfaces (Figure 6). However, with increasing convective cooling intensity, the temperature decreases to the minimum value achieved at $\;Bi_{1}=Bi_{2}≅10$. With a further increase in Biot numbers $Bi_{l}$, l=1,2, the temperature practically does not change.

Increasing the relative thickness of the layers (parameter $d^{*}$) causes an increase in the temperature of the friction surfaces (Figure 7). In the range $0<d^{*}\leq 2$, the temperature of the cermet layer is higher than the temperature of the cast iron layer, while for $d^{*}>2$ the temperature behaviour is reverse. It was found that the temperature of the surface of friction layers with their perfect thermal contact (Bi=100) takes values that are between the corresponding values found for imperfect contact (Bi=1).

**Figure 8:**
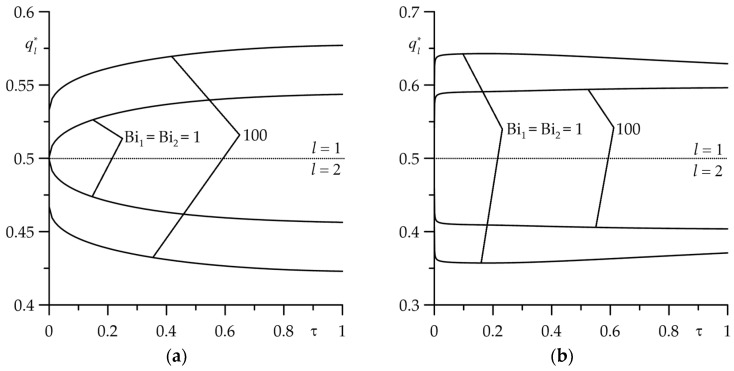
Evolutions of dimensionless heat flux intensities $q_{l}^{*}(\tau )$, l=1,2 for two values of the Biot number: (**a**) Bi=1; (**b**) Bi=100 for $\;Bi_{1}=Bi_{2}=1$ and $\;Bi_{1}=Bi_{2}=100$ at $d^{*}=1$.

During frictional contact, the heat is generated, and the layers heat up. The time profiles of dimensionless heat flux intensities $q_{l}^{*}(\tau )$, l=1,2 (119) absorbed by the layers in the normal direction to the friction surface are presented in Figure 8. Two cases of thermal friction contact were analyzed—imperfect (Bi=1, Figure 8a) and perfect (Bi=100, Figure 8b). Common to both is that the heat flux intensity directed to the cermet layer is higher than that to the cast iron layer. This fact explains the higher temperature on the friction surface of the cermet element compared to the corresponding temperature for the cast iron layer observed in the above figures. The effect of the type of thermal friction contact on the heat flux intensity is significant. At the fixed time instant (the Fourier number τ), in the case of imperfect contact (Bi=1), increasing the intensity of convective cooling on the free surfaces of layers causes an increase in the amount of heat adsorbed by the cermet layer while proportionally reducing the heating of the cast iron layer (Figure 8a). However, in the case of perfect contact, the picture is reversed—with increasing convective cooling intensity, the heat flux intensity adsorbed by the layer made of FMC–11 (ChNMKh) decreases (increases) (Figure 8b). It should also be noted that in all considered cases, boundary condition (11) is satisfied, i.e., $q_{1}^{*}(\tau )+q_{2}^{*}(\tau )=1,\;\tau >0$.

## 7. Conclusions

An analytical model of the frictional heating process for a system of two homogeneous layers was proposed. It takes into account the thermal resistance of the friction surfaces (the thermal contact conductance) and convective heat exchange with the surrounding environment on the free surfaces of the layers. A key element of the model is the parabolic boundary-value problem of heat conductivity. Using the mathematical apparatus of the Laplace integral transform, an exact solution of this problem was obtained. Forms of this solution for the specific values of the input parameters—on surfaces of friction, with perfect friction thermal contact or asymptotic at the initial moments of the heating process were also found.

Based on the above-mentioned solutions, numerical analysis was performed for a cermet (FMC-11) layer sliding uniformly on the surface of a cast iron layer (ChNMKh). This allowed for the investigation of the influence of thermal contact conductance, the intensity of convective cooling, and the relative thickness of the layers on the temperature. The calculation results were presented in dimensionless form. This was intentional since, according to Formulas (8), the transition to dimensional form requires determining the specific friction power, which depends on the operating mode (pressure, velocity, coefficient of friction). However, dimensionless analysis allows for the determination of general temperature dependencies on the input parameters. Such general results include the following:

- For small Fourier numbers, asymptotic solutions closely match the initial temperature evolution obtained from the exact solution, providing a reliable and efficient approach for estimating the temperature field during early-stage heating.
- The presence of a transition stage from the initial to the stationary temperature.
- The temperature jump on the friction surfaces with consideration of the thermal contact conductance. Increasing the thermal contact conductance (reducing the thermal resistance) equalizes the temperature of the friction layers and at Bi≥50 becomes practically the same on both surfaces.
- Reduction in the temperature with increasing intensity of convective cooling.
- The equality of the sum of the heat flux intensities directed from the contact zone to the interior of the layers and the specific power of friction during the heating process.

The results mentioned above are characteristic for models of friction heating based on semi-space/semi-space and layer/semi-space schemes. Obtaining them for the layer–layer system confirms the reliability of the solutions presented above and their suitability for determining temperature when the effective heating depth of the friction pair components is greater than their thickness. An additional advantage of the developed model is the inclusion of convective cooling of the friction system. It should be noted that the obtained solutions for constant specific friction power can be generalized to the case of a changing time profile using Duhamel’s theorem. It should be noted that the specific friction power in braking systems is closely related to the contact pressure. This will have significant application implications, for example, when determining the temperature regime of various types of braking systems. This constitutes our next research goal.

## Data Availability

The original contributions presented in this study are included in the article. Further inquiries can be directed to the corresponding author.

## Nomenclature

Bi	Biot number
d	thickness of the layer, m
$d^{*}$	relative thickness of the layers
f	coefficient of friction
h	heat transfer coefficient, W m^–2^ K^–1^
k	thermal diffusivity, m^2^ s^–1^
$k^{*}$	relative thermal diffusivity of layers
K	thermal conductivity, W m^–1^ K^–1^
$K^{*}$	relative thermal conductivity of layers
p	parameter of the Laplace transform
$p_{0}$	nominal pressure, Pa
$q_{0}$	specific friction power, W m^–2^
q	heat flux intensity, W m^–2^
q*	dimensionless heat flux intensity
t	time, s
T	temperature, °C
$T_{0}$	initial temperature, °C
$V_{0}$	sliding velocity, m s^–1^
z	axial coordinate, m
ε	coefficient of relative thermal activity of friction materials
Θ	temperature rise, °C
$\Theta^{*}$	dimensionless temperature rise
$\Theta_{0}$	temperature scaling coefficient, °C
τ	dimensionless time
ζ	dimensionless axial coordinate
